# Peculiar Characteristics of Arteriovenous Malformations Arising in the Galenic Region

**DOI:** 10.3390/diagnostics10070481

**Published:** 2020-07-15

**Authors:** Hirohisa Yajima, Yuki Shinya, Hirotaka Hasegawa, Masahiro Shin, Keisuke Ueki, Mariko Kawashima, Osamu Ishikawa, Nobuhito Saito

**Affiliations:** 1 Department of Neurosurgery, The University of Tokyo Hospital, Tokyo 1138655, Japan; h.yajima.35@gmail.com (H.Y.); yukishinya6155@gmail.com (Y.S.); SHIN-NSU@h.u-tokyo.ac.jp (M.S.); mrkawashima-tky@umin.ac.jp (M.K.); oishikawa-tky@umin.ac.jp (O.I.); nsaito-tky@umin.net (N.S.); 2 Department of Neurosurgery, Dokkyo Medical University Hospital, Tochigi 3210207, Japan; kueki-tky@umin.ac.jp

**Keywords:** arteriovenous malformations, dural artery feeder, Galenic region, hydrocephalus, vein of Galen

## Abstract

Arteriovenous malformations (AVM) are congenital vascular lesions fed by arterial feeders originating from branches of the internal carotid artery (ICA) or vertebrobasilar artery. We experienced unique AVMs arising in the midline Galenic region, receiving blood supply from the ICA/vertebral artery systems and the external carotid artery system. We retrospectively reviewed data on eight patients who had an AVM arising in the Galenic region and were treated in the University of Tokyo Hospital between 1990 and 2019. The median age at diagnosis was 62 years. Three cases (38%) presented with obstructive hydrocephalus due to aqueduct obstruction caused by an engorged vein of Galen. In all cases, feeders from dural arteries were present and the vein of Galen was the primary drainer. All patients underwent stereotactic radiosurgery. Five patients were followed for > two years; nidus obliteration was confirmed in one, and > 75% shrinkage was confirmed in three, while one patient died due to hemorrhage. Altogether, AVMs arising in the Galenic region are rare and exhibit several peculiar characteristics including the presence of dural feeders, an older age at presentation and presentation with obstructive hydrocephalus.

## 1. Introduction

Brain arteriovenous malformation (AVM) is an arteriovenous shunt disorder consisting of an abnormal vascular network, called “nidus”, that directly connects arteries and veins and is believed to arise during fetal development [1,2,3]. Brain AVM is most commonly diagnosed in patients in their 30 s and 40 s and their symptoms include seizures, headaches, neurological deficit due to ischemia in the adjacent brain area and hemorrhagic strokes [4,5,6,7]. AVMs can arise in various parts of the brain, but the most common locations are the cerebral lobes (70%), basal ganglia and thalamus (15%), cerebellum (7%), corpus callosum (3%) and brainstem (2%) [8].

The Galenic region is one of the deepest locations in the brain with a complicated vascular system [9,10] and is known to accommodate a variety of vascular malformations including dural arteriovenous fistula (DAVF) [11,12,13,14] and vein of Galen aneurysmal malformation (VGAM) [15,16]. Here, we described a unique subtype of AVMs arising in the Galenic region that have not only the above-mentioned features, but also some atypical features.

## 2. Materials and Methods

Data on patients who had AVM and were treated with stereotactic radiosurgery between 1990 and 2019 in our hospital were retrospectively reviewed. Data on patients with AVM in the Galenic region were collated. All patients underwent cerebral angiography, and thus, the detailed vascular structures and clinical information of these cases were analyzed. This study was approved by the institutional review board of our institution (IRB#2231, approved on 12/4/2008). All the patients provided written informed consent for the participation of the study.

## 3. Results

### Radiographic Features

The patients’ radiographic features and presentations are summarized in Table 1. Of note, dural artery feeders were confirmed in all, but one patient in whom external or common carotid angiography was not performed; the tentorial artery was confirmed in six, posterior meningeal artery (via occipital artery) in three, middle meningeal artery in two and inferolateral trunk in two patients. Cerebral artery contributions were also confirmed in all; medial posterior choroidal artery in eight, posterior cerebral artery perforators in six, basilar artery perforators in three and pericallosal artery in two patients. Vein of Galen (VG) to the straight sinus was the exclusive drainage route in all, but two patients in whom drainage of the engorged superior vermian vein into the venous confluence was also a main drainage route. The median nidus volume was 1.7 mL (range, 0.9–12.2 mL). All the niduses were primarily present in the quadrigeminal cistern with an involvement of the surface of the deep brain structures including the pulvinar.

The median age at diagnosis was 62 years (range, 22–76 years). The patients’ initial presentations included hydrocephalus due to aqueduct compression (*n* = 3, 38%), hemorrhage (*n* = 3, 38%), seizure (*n* = 1, 12%) and headache (*n* = 1, 12%). One patient who presented with hydrocephalus developed hemorrhage during the observation period and therefore, four patients experienced hemorrhage before treatment. All AVMs were treated with stereotactic radiosurgery (SRS) using gamma knife (Elekta Instruments AB, Stockholm, Sweden).

**Figure 1:**
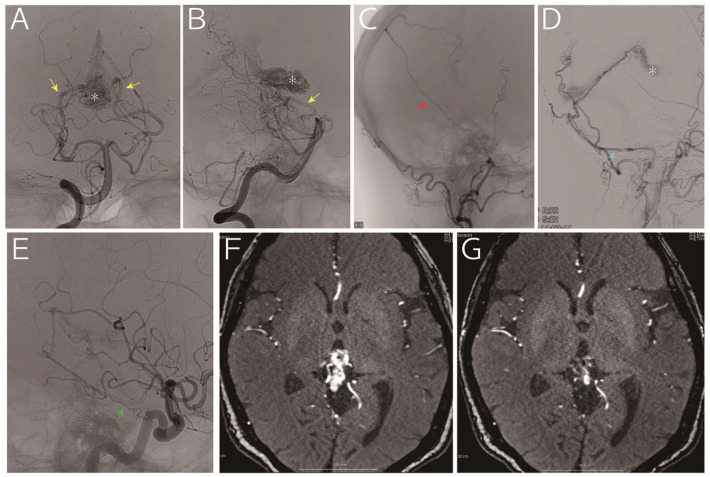
A 63-year-old woman who presented with headache and vertigo without any neurological deficits was referred to us for treatment of an arteriovenous malformation. Digital subtraction angiography (**A** and **B**, vertebral artery; **C**: right external carotid artery; **D**, left external carotid artery; and **E**, left internal carotid artery) indicated a nidus (asterisk) fed by the medial posterior choroidal artery (yellow arrow), posterior cerebral artery perforators, middle meningeal artery (red arrow), posterior meningeal artery (light blue arrow, via the occipital artery) and tentorial artery (green arrow). The drainage vein was the vein of Galen. Considering that a part of the nidus was embedded in the thalamus (**F**, time-of-flight magnetic resonance imaging), we decided to treat using gamma knife, and the images obtained 30 months later showed remarkable reduction in the nidus without any sings of hemorrhage (**G**, time-of-flight magnetic resonance imaging).

**Figure 2:**
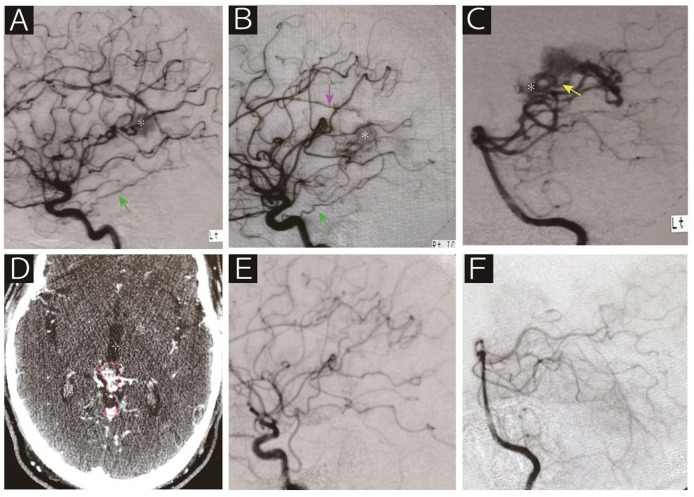
A 62-year-old man who presented with epilepsy was referred to us for radiosurgical treatment for an arteriovenous malformation. Digital subtraction angiography (**A**, left internal carotid artery; **B**, right internal carotid artery; **C**, left vertebral artery) showed that the nidus (asterisk) was supplied by the bilateral tentorial artery (green arrow), right callosal artery (purple arrow), medial posterior choroidal artery (yellow arrow) and posterior cerebral artery perforators. Contrasted computed tomography on the day of radiosurgery revealed the nidus adjacent to the vein of Galen (**D**). Note that the red line indicates the radiosurgical target. Angiographic obliteration was confirmed 40 months after radiosurgery without any deficits (**E**, right common carotid artery; **F**, left vertebral artery). The patient has been following a stable course over 10 years.

**Figure 3:**
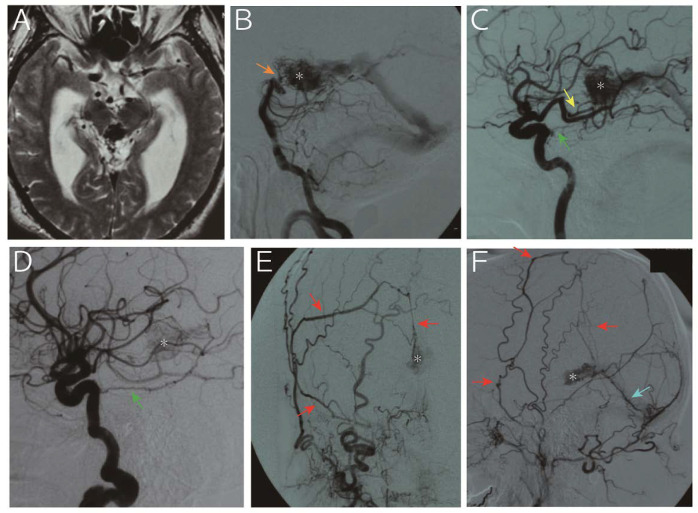
A 65-year-old man was referred to us for progressive headache and gait disturbance. Imaging study revealed obstructive hydrocephalus due to aqueduct stenosis caused by an abnormal vascular structure in the pineal region (**A**, T2 weighted image). Digital subtraction angiography (**B**, vertebral artery; **C**, left internal carotid artery; **D**, right internal carotid artery; **E**, right external carotid artery; **F**, left external carotid artery) showed that the nidus (asterisk) was fed by the medial posterior choroidal artery (yellow arrow), basilar artery perforators (orange arrow), bilateral tentorial artery (green arrow), middle meningeal artery (red arrows) and posterior meningeal artery (light blue arrow) and drained into the vein of Galen. Following endoscopic third ventriculostomy for hydrocephalus, the arteriovenous malformation was treated with gamma knife. However, the nidus persisted with slight size reduction 25 months thereafter, and the patient refused further treatment due to senility. The patient developed subarachnoid hemorrhage due to a rupture of the malformation 153 months after the treatment and died.

The median follow-up period after SRS was 43 months (range, 0–255 months). Nidus obliteration was confirmed in one patient and > 75% shrinkage was confirmed in three. Successful shrinkage was confirmed in two patients at follow-up (for 7 and 38 months) and immediately after SRS in two patients. Hydrocephalus was treated using a ventriculoperitoneal shunt in one patient and endoscopic third ventriculostomy in another, with good control in each. As of now, seven patients were neurologically stable, whereas one patient with >75% shrinkage in the nidus developed a fatal hemorrhage at 153 months after the SRS. Detailed case illustrations are shown in Figure 1, Figure 2 and Figure 3.

## 4. Discussion

The presented vascular malformations were diagnosed as AVM due to the presence of intra-axial nidus [2,17]. Nevertheless, in addition to the common features of AVMs, AVMs arising in the Galenic region have some specific features: high possibility of hydrocephalus, presence of dural artery feeders and relatively late onset of disease. In AVMs of the Galenic region, the enlarged VG and nidus caused compression of the mesencephalic aqueduct, resulting in obstructive hydrocephalus. This mechanism, in addition to the vascular angioarchitecture, is reminiscent of VGAM. VGAM is an arteriovenous fistula of the median prosencephalic vein (a precursor of the vein of Galen) without a nidus occurring during gestation [15,16]. VGAM is usually detected in neonates and children and is clearly different from the malformation in our cases. Adult-onset VGAM is rare, but possible, though it is characterized by a direct fistula without a nidus [18,19,20] David et al. indicated that the persistent falcine sinus and thrombosed VGAM are angiographic features of an adult VGAM, which were not observed in the present series [21].

We do not completely understand the reason for the late onset of AVMs in the Galenic region. Since they rarely cause seizure, they never get discovered until they develop hemorrhage or hydrocephalus except for incidental discovery. The presence of the VG as their direct drainer may lead to generous capacity of venous drainage, making early accumulation of hemodynamic stress and the resultant rupture less likely in the youth. Unlike VGAM, Galenic AVM has a nidus that buffers high inflow pressure and thus would retard the process of aneurysmal dilatation of the VG, possibly leading to the senior onset of hydrocephalus.

Presence of dural artery feeders and middle-age onset are common characteristics of DAVF. DAVFs especially in the falco–tentorial regions may have drainage via the VG as well as pial feeders such as the artery of Davidoff–Schechter and dural branch of the superior cerebellar artery, making it difficult to distinguish them from Galenic AVMs [22,23]. However, by definition, such DAVFs have their shunts in the dural membranes of the sinus or tentorium and should not have any cisternal components [24,25,26,27,28]. In the enclosed AVMs, all cases apparently had a nidus that was located in the cisternal space along with the VG with an involvement of the brain surface, and thus were considered as AVM.

Presence of a deep nidus involving the thalamus and/or the midbrain prompted us to use SRS. Our cases suggest the efficacy of this method as five out of six patients who were followed up for >2 years showed obliteration or significant shrinkage of the nidus. However, the rate of obliteration may be lower than AVMs in the other regions [29]. Since the risk of rupture can be completely avoided only after the nidus is completely obliterated, further long-term observation is mandatory [30,31]. Endovascular embolization may also be feasible considering that the malformation usually has prominent dural feeders; however, meticulous care should be taken as a high-flow shunt draining into the VG is often present and severe complications are possible if this shunt is occluded.

This study had several limitations. First, the lack of microscopic findings may decrease the robustness of this report. The location of the nidus in the Galenic region is not wedged into the brain like normal AVMs. However, given the detailed radiographic findings, there is a high chance that the lesions had an intra-axial component and should be classified as AVMs. They could be similar to the intraventricular AVMs in the quadrigeminal cistern and involve the cerebral parenchyma. Second, due to the lack of long-term observation data, further research would be desirable to determine the optimal treatment strategy. Despite these limitations, the peculiar clinical and radiographic features of the enclosed AVMs are worth reporting.

## 5. Conclusions

We report eight cases of AVM arising in the Galenic region that harbored both cerebral and dural artery feeders and drained into the VG. These patients often presented with hydrocephalus due to compression of the aqueduct caused by engorgement of the draining vein. These AVMs may have some features that can be shared with VGAM and DAVF in the tentorial edge; however, given its distinct characteristics, AVM arising in the Galenic region should be considered as a distinguished AVM subtype.

## Figures and Tables

**Table 1:** Patient clinical and angioarchitectural features.

No.	Age *, Sex	Initial Presentation	Volume	Cerebral Artery Feeders	Dural Artery Feeders	Drainers	Follow-Up Period	Current Status
1	65, M	Hydrocephalus	9.4 mL	MPChA, BA perforators	Tentorial a., MMA, PMA	VG	153 mo	Died due to hemorrhage
2	62, M	Seizure	1.4 mL	MPChA, PCA perforators, pericallosal a.	Tentorial a.	VG	255 mo	Alive, nidus obliteration
3	68, M	Hydrocephalus	0.9 mL	MPChA, PCA perforators	N/A	VG	137 mo	Alive, nidus shrinkage
4	31, F	Hydrocephalus and hemorrhage †	12.2 mL	MPChA, PCA perforators	Tentorial a (N/A for the other ECA feeders)	VG	38 mo	Alive, nidus shrinkage
5	63, F	Headache	2.3 mL	MPChA, PCA perforators, BA perforators	Tentorial a, MMA, PMA	VG	47 mo	Alive, nidus shrinkage
6	62, M	Hemorrhage	1.2 mL	MPChA, BA perforators	Tentorial a, ILT	VG, superior vermian vein	23 mo	Alive, nidus shrinkage
7	55, M	Hemorrhage from flow-related AN	1.6 mL	MPChA, pericallosal a.	ILT, PMA	VG, superior vermian vein	12 mo	Alive, nidus shrinkage
8	76, M	Hemorrhage	1.7 mL	MPChA, PCA perforators	Tentorial a.	VG	4 mo	Alive

AVM = arteriovenous malformation; BA = basilar artery; ECA = external carotid artery; ILT = inferolateral trunk; MMA = middle meningeal artery; mo = months; MPChA = medial posterior choroidal artery; N/A = not assessed; PCA = posterior cerebral artery; PMA = posterior meningeal artery; Sup. = superior; VG = vein of Galen, AN = aneurysm. * Age at diagnosis. † Hemorrhage developed after 10 years of observation period since the initial presentation when the patient presented with hydrocephalus and treated with shunt.
